# BRAF Mutation Affects Intratumor Heterogeneity in Papillary Thyroid Carcinoma

**DOI:** 10.1155/ancp/8843563

**Published:** 2026-05-18

**Authors:** Ying-er Feng, Cun-jie Wang

**Affiliations:** ^1^ Ultrasound Nursing, The Second Affiliated Hospital Zhejiang University School of Medicine, Hangzhou, 310009, Zhejiang, China, z2hospital.com; ^2^ General Medicine, The Second Affiliated Hospital Zhejiang University School of Medicine, Zheda Campus, Hangzhou, 310027, Zhejiang, China, z2hospital.com

**Keywords:** BRAF mutation, intratumor heterogeneity, papillary thyroid carcinoma, thyroid differentiation score

## Abstract

**Background:**

Intratumor heterogeneity (ITH) plays an important role in patients’ clinical outcomes. The prognostic impact of ITH and its influencing factors is unclear in papillary thyroid carcinoma (PTC), which deserves further investigation.

**Methods:**

The Mutation Annotation Format (MAF) and clinical features were collected from The Cancer Genome Atlas Thyroid Cancer (TCGA‐THCA) cohort. We first assessed the influence of ITH on the prognosis of patients. We used the Mutant Allele Tumor Heterogeneity score to evaluate and represent ITH. Then we explored the potential factors associated with ITH. Finally, we predicted possible pathways involved in ITH.

**Results:**

Among 4 prognostic outcomes, higher ITH was mainly related to poor disease‐free interval (DFI) (HR = 2.64, *p* = 0.01), and ITH had potential value for predicting DFI. Further, we identified BRAF mutation and thyroid differentiation score (TDS) as key factors independently influencing ITH (all *p*  < 0.05), especially TDS, which had a favorable ability and obtained good net benefit in predicting ITH. TDS maintained a stable negative effect on ITH. In contrast, BRAF mutation positively correlated with ITH in univariable regression (β = 0.196). Through performing sensitivity, regression, subgroup, interaction effect, and mediation analyses, we identified that TDS played a suppression effect in the impact of BRAF mutation on ITH. Finally, we revealed that the propionate metabolism pathway was most strongly associated with ITH.

**Conclusions:**

ITH is associated with DFI in patients with PTC and deserves more attention. TDS and BRAF mutation are the key influencing factors of ITH, but TDS exerts a suppression effect on the impact of BRAF mutation on ITH.

## 1. Introduction

Papillary thyroid carcinoma (PTC) is the most common malignant thyroid tumor, accounting for over 80% of all thyroid cancers (THCAs). Over the past few decades, the incidence of PTC has shown a significant upward trend, making it one of the fastest‐growing malignancies worldwide [1–4]. While the overall prognosis for PTC is favorable, with a 5‐year survival rate exceeding 95%, some patients exhibit highly variable biological behaviors, including local invasion, lymph node metastasis, and distant metastasis, which lead to worse clinical outcomes [5, 6].

Recently, intratumor heterogeneity (ITH) has emerged as a key focus in cancer research. ITH refers to the genetic, transcriptomic, and epigenetic differences among cancer cells within the same tumor, playing a crucial role in tumor initiation, progression, drug resistance, and recurrence [7]. ITH in different aspects is interrelated. For instance, a study by Touat et al. [8] demonstrated that in gliomas, ITH is significantly correlated with tumor microsatellite instability (MSI). In breast cancer, the dynamic heterogeneity of the immune stroma pattern affects both malignant tumor cells and immune cells in the tumor microenvironment, including tumor‐infiltrating lymphocytes [9, 10]. Tumor metabolic heterogeneity can also promote tumor epigenetic heterogeneity [11]. These various forms of heterogeneity contribute to the tumor’s evolutionary potential, thereby promoting tumor development and the occurrence of poor patient prognosis [12].

However, the relationship between ITH and prognosis in PTC remains unclear. Therefore, the present study, based on The Cancer Genome Atlas (TCGA) database, analyzed the impact of ITH on the disease prognosis of PTC patients, including disease‐free interval (DFI), progression‐free survival (PFS), overall survival (OS), and disease‐specific survival (DSS). The results revealed a significant association between ITH and DFI in PTC. Further investigation identified key clinical features influencing ITH, with a focus on the role and interaction of v‐Raf murine sarcoma viral oncogene homolog B1 (BRAF) mutations and thyroid differentiation score (TDS). Lastly, pathway analysis revealed a significant correlation between ITH and the propionate metabolism pathway, exploring its potential regulatory role in the formation of ITH. This study provides new directions for the personalized treatment and precision medicine of PTC in the future.

## 2. Materials and Methods

### 2.1. Data Sources

The data for this study were obtained from TCGA, a large‐scale cancer genomics project launched in 2006 by the National Cancer Institute (NCI) and the National Human Genome Research Institute (NHGRI). TCGA integrates multi‐omics data—including genomic, transcriptomic, epigenomic, and proteomic information—to elucidate the molecular characteristics of cancers and improve their diagnosis, treatment, and prevention.

We collected survival data, clinical information, and single‐nucleotide variant (SNV) mutation annotation files of 507 patients with THCA from the TCGA official website, covering the period from 2011 to 2015. After excluding 5 patients without SNV mutation annotation files, a total of 502 patients were included in the study. The analysis of SNV mutation annotation files was performed following the methods of Colaprico et al. [13], and data were downloaded using the TCGA *biolinks* R/Bioconductor package with the Masking workflow.

### 2.2. Somatic Mutation Analysis and Measurement of Heterogeneity

The R/Bioconductor package Maftools [14] was used to comprehensively analyze somatic mutations in PTC. Tumor heterogeneity, as represented by the Mutant Allele Tumor Heterogeneity (MATH) score, was calculated based on the variant allele frequency (VAF) of all mutations within the tumor. The inferHeterogeneity function in Maftools clusters VAF data using the *mclust* algorithm to infer clonality. Each sample was assigned a MATH score, reflecting ITH: higher MATH scores indicate higher heterogeneity [15]. In this study, the MATH score was used to assess ITH.

### 2.3. Clinical Value Analysis on ITH in PTC

The clinical value of ITH was assessed from the prognostic effect, prediction performance, and clinical net benefit. The “maxstat” R package was used to determine the optimal cutoff value for MATH, dividing patients into high‐ and low‐ITH groups. Kaplan–Meier (KM) survival curves were generated to explore the impact of ITH on different prognostic outcomes, including DFI, PFI, OS, and DSS. Survival differences between the groups were evaluated using the log‐rank test with a significance threshold of *p*  < 0.05. Receiver operating characteristic (ROC) and decision curve analysis (DCA) were used to assess the performance of ITH in predicting patient outcomes.

### 2.4. Influencing Factor Analysis Associated With ITH

In this study, the influencing factors selected for analysis included the aspects of baseline clinical characteristics of patients, mutation level, tumor purity, immune microenvironment, and differentiation level. The baseline clinical characteristics contained 28 variables. The mutation level was reflected by the MSI. The immune microenvironment was assessed by the infiltration abundance of 10 kinds of immune cells, which was calculated by the Cibersort algorithm. The differentiation level of PTC was evaluated by TDS. TDS is a quantitative metric derived from the average Log2 (fold‐change) in expression of 16 genes related to thyroid function (DIO1, DIO2, DUOX1, DUOX2, FOXE1, GLIS3, NKX2‐1, PAX8, SLC26A4, SLC5A5, SLC5A8, TG, THRA, THRB, TPO, and TSHR). This score indicates the degree to which tumor cells transcriptomically resemble normal thyroid tissue. Elevated TDS values correspond to a well‐differentiated phenotype, whereas reduced TDS signifies dedifferentiation and increased malignant potential.

We first compared the differences in these aspects between the ITH high and low groups. Continuous clinical variables that did not follow a normal distribution were compared using the Mann–Whitney *U* test and expressed as median (Q25, Q75). Categorical variables were compared using the chi‐square test and expressed as *n* (%). Variables showing significant differences were analyzed for collinearity to remove those with multicollinearity. Variables with a variance inflation factor (VIF) greater than 5 are considered to have collinearity. Univariate linear regression was then performed to identify clinical factors significantly associated with ITH. Independent correlation analyses were conducted to evaluate the relationships between ITH and the selected clinical variables. This process identified BRAF mutation and TDS as key factors affecting ITH. ROC and DCA were then used to evaluate their predictive efficacy on ITH.

### 2.5. Role of TDS on the Association Between BRAF Mutation and ITH

The impact of BRAF mutation on ITH was opposite in univariable and multivariable regression analyses. Therefore, we next explored the potential reasons. A linear regression model was first used to investigate the relationship between ITH and BRAF mutation by separately adjusting for other variables. The *t*‐test was used to compare ITH across subgroups and revealed that TDS influenced the association between BRAF mutation and ITH. Subgroup logistic regression and linear regression analyses were employed to validate the impact of TDS and BRAF mutation on ITH. Mediation and interaction analyses were conducted to explore the specific role of TDS in the relationship between BRAF mutation and ITH. Statistical significance was set at *p*  < 0.05.

### 2.6. Exploration of ITH‐Related Pathways

Single‐sample Gene Set Enrichment Analysis (ssGSEA) was performed to calculate pathway scores of 186 Kyoto Encyclopedia of Genes and Genomes (KEGG) pathways. Independent sample *t*‐tests were conducted to compare KEGG scores between the high‐ and low‐ITH groups, identifying pathways with significant differences. Correlation analyses were then performed to determine pathways significantly associated with ITH. Multivariate linear regression was subsequently applied to identify key pathways influencing ITH. Statistical significance was set at *p*  < 0.05.

## 3. Results

### 3.1. Relationship Between ITH and Prognosis in PTC Patients

**Figure 1:**
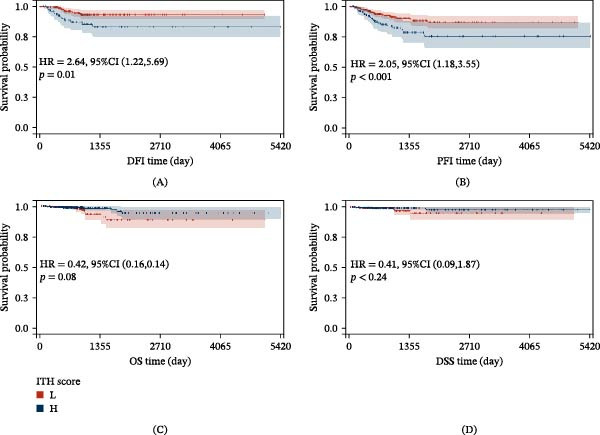
Survival analysis of ITH score in different prognoses for patients with PTC. (A) DFI, (B) PFI, (C) OS, and (D) DSS. According to the optimal cutoff value of ITH, the population was divided into high and low groups. Abbreviations: CI, confidence interval; DFI, disease‐free interval; DSS, disease‐specific survival; HR, hazard ratio; ITH, intratumoral heterogeneity; OS, overall survival; PFI, progression‐free survival; PTC, papillary thyroid carcinoma.

**Figure 2:**
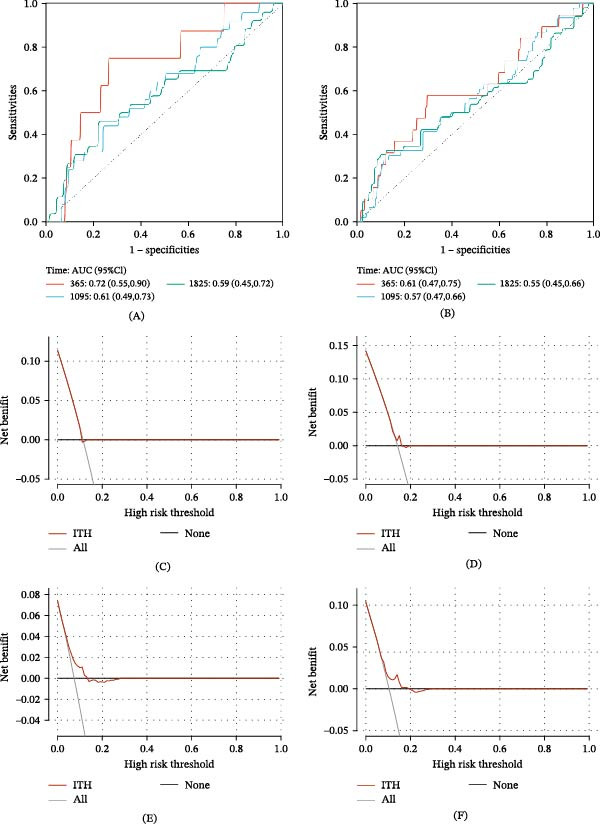
ROC and DCA analysis evaluated the prognosis prediction efficiency of ITH in patients with PTC. (A) Time‐dependent ROC curves of DFI according to ITH. (B) Time‐dependent ROC curves of PFI according to ITH. (C) DCA curves of DFI according to ITH in 1‐year survival. (D) DCA curves of PFI according to ITH in 1‐year survival. (E) DCA curves of DFI according to ITH in all survival. (F) DCA curves of PFI according to ITH in all survival. Abbreviations: AUC, area under the curve; CI, confidence interval; DCA, decision curve analysis; DFI, disease‐free interval; ITH, intratumoral heterogeneity; PFI, progression‐free survival; PTC, papillary thyroid carcinoma; ROC, receiver operating characteristic curve.

We explored the clinical significance of ITH on the prognosis of PTC patients through KM survival analysis. KM results demonstrated that ITH significantly impacted DFI and PFI (*p*  < 0.05, Figure 1A,B), with patients in the low‐ITH group exhibiting higher survival rates. However, no significant impact was observed on OS or DSS (*p*  > 0.05, Figure 1C,D). Time‐dependent ROC analysis showed that the area under the curve (AUC) for ITH in predicting 1‐, 3‐, and 5‐year DFI was 0.72, 0.61, and 0.59, respectively (Figure 2A). Similarly, the AUC values for predicting 1‐, 3‐, and 5‐year PFI were 0.61, 0.57, and 0.55, respectively (Figure 2B). These findings suggest that ITH can predict DFI and PFI, with the best predictive performance for 1‐year DFI or PFI. Further DCA revealed no clinical net benefit for ITH in predicting 1‐year DFI or PFI (Figure 2C,D). However, for whole DFI, a net clinical benefit was observed when the threshold probability was within the range of 0.10–0.16 (Figure 2E). For PFI, the threshold range was 0.12–0.20 (Figure 2F). These results suggested that ITH is most effective in predicting DFI.

### 3.2. Screening Clinical Features Associated With ITH

Given the prognostic value of ITH, we investigated clinical factors influencing ITH. Patients were divided into high‐ and low‐ITH groups based on the optimal cutoff value for MATH score, and clinical characteristics between the two groups were compared. Significant differences were found in tumor width, tumor length, tumor depth, T stage, extrathyroidal extension, lymph node ratio, number of positive lymph nodes, TDS, staging, BRAF mutation, histological type, regulatory T cells (Tregs), follow‐up radiation therapy, postsurgical thyroid gland carcinoma status, and personal medical history (*p*  < 0.05, Tables 1,2). However, tumor width exhibited collinearity (VIF = 10.935, Table 3) and was excluded from further analyses.

**Table 1:** Baseline features (continuous variable) of patients with PTC.

Variables	Miss	Low‐ITH group (*n* = 366)	High‐ITH group (*n* = 136)	*U*	*p*
Tumor purity	43	0.690 [0.600, 0.820]	0.670 [0.590, 0.820]	0.381	0.703
TDS	114	0.318[−0.502, 1.170]	−1.089 [−1.470, −0.640]	10.888	<0.001
MSI	12	0.309 [0.295, 0.321]	0.311 [0.297, 0.321]	−0.364	0.716
Age at initial pathologic diagnosis	0	46.000 [36.000, 58.000]	47.000 [34.000, 60.000]	0.136	0.892
Number of detected lymph nodes	114	5.000 [2.000, 14.000]	7.000 [3.000, 17.000]	−1.935	0.052
Number of positive lymph nodes	116	1.000 [0.000, 4.000]	2.000 [0.000, 6.000]	−2.194	0.022
Lymph node ratio	119	0.091 [0.000, 0.385]	0.250 [0.000, 0.519]	−2.569	0.007
Depth of tumor	99	1.500 [1.000, 2.000]	1.800 [1.300, 2.500]	−3.065	0.002
Length of tumor	29	2.500 [1.500, 3.600]	3.000 [2.000, 4.400]	−2.673	0.007
Width of tumor	77	2.000 [1.300, 2.700]	2.500 [1.600, 3.200]	−3.708	<0.001
Course of disease	1	1.000 [1.000, 4.000]	1.000 [1.000, 4.000]	−0.592	0.533
B cells memory	143	0.052 [0.023, 0.087]	0.042 [0.020, 0.071]	1.621	0.105
Plasma cells	97	0.041 [0.017, 0.085]	0.051 [0.018, 0.106]	−1.183	0.237
T cells CD8	8	0.119 [0.073, 0.170]	0.108 [0.061, 0.154]	1.873	0.061
T cells regulatory (Tregs)	103	0.020 [0.008, 0.035]	0.031 [0.017, 0.047]	−4.560	<0.001
NK cells activated	15	0.051 [0.032, 0.067]	0.049 [0.034, 0.071]	−0.537	0.591
Monocytes	50	0.032 [0.015, 0.061]	0.035 [0.016, 0.061]	−0.759	0.448
Macrophages M1	110	0.025 [0.013, 0.043]	0.028 [0.007, 0.055]	−0.179	0.858
Macrophages M2	3	0.282 [0.201, 0.358]	0.255 [0.180, 0.352]	1.719	0.086
Dendritic cells activated	290	0.013 [0.005, 0.027]	0.019 [0.005, 0.034]	−1.700	0.089
T cells CD4 memory activated	295	0.012 [0.006, 0.021]	0.014 [0.007, 0.030]	−1.697	0.090
Dose of iodine 131	228	100.000 [75.300, 148.800]	104.100 [80.000, 150.000]	−0.963	0.335

Abbreviations: ITH, intratumoral heterogeneity; MSI, microsatellite instability; PTC, papillary thyroid carcinoma; TDS, thyroid differentiation score.

**Table 2:** Baseline features (categorical variables) of patients with PTC.

Variables	Miss	Low‐ITH (*n* = 366)	High‐ITH (*n* = 136)	*χ* ^2^	*p*
BRAF mutation, *n*(%)	18	—	—	24.021	<0.001
No	—	168 (47.863)	31 (23.308)	—	—
Yes	—	183 (52.137)	102 (76.692)	—	—
Gender, *n*(%)	0	—	—	0.104	0.747
Male	—	97 (26.503)	38 (27.941)	—	—
Female	—	269 (73.497)	98 (72.059)	—	—
Histological type, *n*(%)	9	—	—	28.037	<0.001
Classical/usual	—	244 (67.967)	112 (83.582)	—	—
Follicular	—	94 (26.184)	7 (5.224)	—	—
Tall cell	—	21 (5.850)	15 (11.194)	—	—
T stage, *n*(%)	2	—	—	6.663	0.010
T1+T2	—	236 (64.835)	71 (52.206)	—	—
T3+T4	—	128 (35.165)	65 (47.794)	—	—
N, *n*(%)	0	—	—	11.804	<0.001
N0	—	184 (50.273)	45 (33.088)	—	—
N1	—	182 (49.727)	91 (66.912)	—	—
M, *n*(%)	1	—	—	1.706	0.192
M0	—	199 (54.521)	83 (61.029)	—	—
M1	—	166 (45.479)	53 (38.971)	—	—
Stage, *n*(%)	2	—	—	5.43	0.020
Early	—	254 (69.589)	79 (58.519)	—	—
Advanced	—	111 (30.411)	56 (41.481)	—	—
Race, *n*(%)	103	—	—	3.77	0.052
Other	—	263 (92.281)	98 (85.965)	—	—
Hispanic	—	22 (7.719)	16 (14.035)	—	—
Extra thyroid carcinoma present extension status, *n*(%)	18	—	—	8.684	0.013
None	—	254 (71.751)	77 (59.231)	—	—
Minimal (T3)	—	90 (25.424)	44 (33.846)	—	—
Moderate/Advanced (T4a)	—	10 (2.825)	9 (6.923)	—	—
Follow‐up radiation therapy, *n*(%)	59	—	—	5.639	0.018
No	—	125 (38.462)	31 (26.271)	—	—
Yes	—	200 (61.538)	87 (73.729)	—	—
Postsurgical procedure assessment thyroid gland carcinoma status, *n*(%)	169	—	—	12.282	<0.001
No	—	233 (91.732)	61 (77.215)	—	—
Yes	—	21 (8.268)	18 (22.785)	—	—
New event tissue, *n*(%)	91	—	—	2.753	0.097
No	—	277 (93.581)	102 (88.696)	—	—
Yes	—	19 (6.419)	13 (11.304)	—	—
Lymph node preoperative assessment diagnostic imaging type, *n*(%)	133	—	—	2.94	0.086
Ultrasound alone	—	211 (79.026)	72 (70.588)	—	—
Nonsingle ultrasound	—	56 (20.974)	30 (29.412)	—	—
Personal medical history, *n*(%)	84	—	—	7.935	0.019
Normal	—	195 (63.725)	84 (75.000)	—	—
Nodular hyperplasia	—	59 (19.281)	9 (8.036)	—	—
Lymphocytic thyroiditis	—	52 (16.993)	19 (16.964)	—	—
Primary neoplasm focus type, *n*(%)	10	—	—	2.021	0.155
1	—	186 (52.101)	80 (59.259)	—	—
>1	—	171 (47.899)	55 (40.741)	—	—
Primary thyroid gland neoplasm location anatomic site, *n*(%)	6	—	—	1.41	0.494
Left lobe	—	123 (33.978)	52 (38.806)	—	—
Right lobe	—	161 (44.475)	52 (38.806)	—	—
Isthmus/bilateral	—	78 (21.547)	30 (22.388)	—	—
Radiations radiation measure of response, *n*(%)	311	—	—	1.617	0.203
No	—	18 (14.286)	14 (21.538)	—	—
Yes	—	108 (85.714)	51 (78.462)	—	—
Type of radiotherapy, *n*(%)	232	—	—	1.549	0.213
Systemic alone	—	164 (91.620)	79 (86.813)	—	—
Nonsystemic alone	—	15 (8.380)	12 (13.187)	—	—
Residual tumor, *n*(%)	62	—	—	1.104	0.293
No	—	286 (88.272)	98 (84.483)	—	—
Yes	—	38 (11.728)	18 (15.517)	—	—

Abbreviations: BRAF, v‐Raf murine sarcoma viral oncogene homolog B1; ITH, intratumoral heterogeneity; PTC, papillary thyroid carcinoma; TDS, thyroid differentiation score.

**Table 3:** Collinearity analysis.

Variables	VIF
Width of tumor	10.935
Length of tumor	6.164
Depth of tumor	5.934
T stage	3.499
Extra thyroid carcinoma presents extension status	3.443
*N*	2.574
Lymph node ratio	2.216
Number of positive lymph nodes	1.685
TDS	1.598
Stage	1.559
BRAF mutation	1.539
Histological type	1.528
T cells regulatory (Tregs)	1.269
Follow‐up radiation therapy	1.232
Postsurgical procedure assessment thyroid gland carcinoma status	1.158
Personal medical history	1.100

Abbreviations: BRAF, v‐Raf murine sarcoma viral oncogene homolog B1; TDS, thyroid differentiation score; VIF, variance inflation factor.

Univariate linear regression analysis revealed that TDS, lymph node ratio, number of positive lymph nodes, tumor depth, tumor length, Tregs, BRAF mutation, histological type (follicular and tall cell), T stage (T1 + T2 vs. T3 + T4), advanced stage, postsurgical thyroid carcinoma status, follow‐up radiation therapy, extrathyroidal extension status (T3 and T4a), and personal medical history (nodular hyperplasia and lymphocytic thyroiditis) were linearly associated with ITH (*p*  < 0.05, Table 4). To address missing data, we performed imputation. Both original and imputed datasets underwent multivariable linear regression analyses, and only TDS and BRAF mutation independently and negatively correlated with ITH (*p*  < 0.05, Table 5).

**Table 4:** Association between several factors and ITH by univariate linear regression analysis.

Variables	*N*	β [95% CI]	*p*
TDS	388	−0.189 [−0.204,−0.175]	<0.001
Lymph node ratio	386	0.003 [−0.001,0.007]	0.174
Number of positive lymph nodes	383	0.109 [0.025,0.192]	0.011
Depth of tumor	403	0.048 [0.019,0.077]	0.001
Length of tumor	473	0.02 [0.005,0.034]	0.008
T cells regulatory (Tregs)	399	2.559 [1.44,3.678]	<0.001
BRAF mutation			
No	199	Ref	Ref
Yes	285	0.196 [0.150,0.242]	<0.001
Histological type			
Classical/usual	356	Ref	Ref
Follicular	101	−0.208 [−0.266,−0.150]	<0.001
Tall cell	36	0.072 [−0.017,0.162]	0.113
T stage			
T1 + T2	307	Ref	Ref
T3 + T4	193	0.082 [0.033,0.131]	0.001
N			
N0	229	Ref	Ref
N1	273	0.115 [0.068,0.162]	<0.001
Stage			
Early	333	Ref	Ref
Advanced	167	0.07 [0.020,0.121]	0.006
Extra thyroid carcinoma present extension status			
None	331	Ref	Ref
Minimal (T3)	134	0.078 [0.024,0.132]	0.005
Moderate/advanced (T4a)	19	0.186 [0.062,0.311]	0.003
Postsurgical procedure assessment thyroid gland carcinoma status			
No	294	Ref	Ref
Yes	39	0.125 [0.036,0.214]	0.006
Follow‐up radiation therapy			
No	156	Ref	Ref
Yes	287	0.086 [0.034,0.139]	0.001
Personal medical history			
Normal	279	Ref	Ref
Nodular hyperplasia	68	−0.148 [−0.219,−0.076]	<0.001
Lymphocytic thyroiditis	71	−0.032 [−0.102,0.038]	0.368

*Note*: Lymph node ratio is the number of positive lymph nodes to the number of detected lymph nodes.

Abbreviations: BRAF, v‐Raf murine sarcoma viral oncogene homolog B1; CI, confidence interval; TDS, thyroid differentiation score.

**Table 5:** Association between several factors and ITH by multivariate linear regression analysis.

Variables	Raw data				Interpolation data			
	Coef	Lower	Upper	*p*	Coef	Lower	Upper	*p*
TDS	−0.200	−0.238	−0.162	<0.001	−0.203	−0.226	−0.180	<0.001
Lymph node ratio	0.001	−0.005	0.008	0.681	−0.003	−0.006	0.001	0.163
Number of positive lymph nodes	−0.059	−0.193	0.075	0.387	−0.066	−0.15	0.019	0.128
Depth of tumor	0.045	−0.02	0.109	0.176	0.011	−0.021	0.044	0.495
Length of tumor	−0.023	−0.062	0.017	0.263	0.004	−0.014	0.022	0.651
T cells regulatory (Tregs)	−0.322	−1.832	1.189	0.677	0.919	0.074	1.763	0.034
BRAF mutation	−0.093	−0.172	−0.013	0.024	−0.075	−0.120	−0.029	0.001
Histological type (follicular)	−0.027	−0.126	0.071	0.587	−0.013	−0.062	0.035	0.592
Histological type (tell cell)	0.004	−0.096	0.103	0.939	−0.016	−0.086	0.054	0.65
T3 + T4	0.097	−0.021	0.214	0.111	0.042	−0.023	0.108	0.208
N1	0.009	−0.082	0.101	0.845	0.045	−0.012	0.102	0.119
Advanced stage	0.029	−0.045	0.103	0.444	−0.023	−0.067	0.021	0.301
Postsurgical procedure assessment thyroid gland carcinoma status (yes)	−0.026	−0.13	0.079	0.629	−0.097	−0.161	−0.033	0.003
Follow‐up radiation therapy (yes)	0.053	−0.015	0.122	0.130	−0.133	−0.240	−0.025	0.016
Extra thyroid carcinoma present extension status (minimal [T3])	−0.118	−0.239	0.002	0.058	0.008	−0.053	0.069	0.796
Extra thyroid carcinoma present extension status (moderate/advanced [T4a])	0.044	−0.296	0.384	0.799	0.028	−0.011	0.067	0.166
Personal medical history (nodular hyperplasia)	−0.036	−0.113	0.04	0.357	−0.037	−0.082	0.008	0.109
Personal medical history (lymphocytic thyroiditis)	0.026	−0.04	0.092	0.445	0.013	−0.037	0.063	0.608

Abbreviations: BRAF, v‐Raf murine sarcoma viral oncogene homolog B1; TDS, thyroid differentiation score.

**Figure 3:**
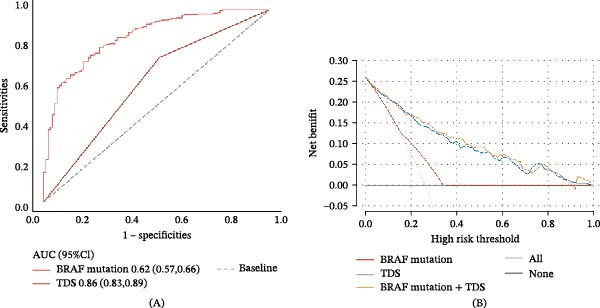
ROC and DCA analysis evaluated the prediction efficiency of key variables in ITH. (A) ROC curves of ITH according to BRAF mutation/TDS. (B) DCA curves of ITH according to BRAF mutation/TDS. Abbreviations: AUC, area under the curve; BRAF, v‐Raf murine sarcoma viral oncogene homolog B1; CI, confidence interval; DCA, decision curve analysis; ITH, intratumoral heterogeneity; ROC, receiver operating characteristic curve; TDS, thyroid differentiation score.

ROC analysis showed that the AUC values for the BRAF mutation and TDS in predicting ITH were 0.62 and 0.86, respectively (Figure 3A). Sensitivity, specificity, and accuracy were 0.75, 0.49, and 0.56 for BRAF mutation and 0.81, 0.75, and 0.77 for TDS. DCA indicated that BRAF mutation predicted ITH with clinical net benefit within a threshold probability of 0.18–0.35, while TDS demonstrated clinical net benefit within a broader range of 0.02–0.96. Combining BRAF mutation and TDS achieved clinical net benefit across the entire threshold probability range of 0.01–1.00 (Figure 3B).

### 3.3. Effects of Key Clinical Features on ITH

Our findings identified BRAF mutation and TDS as key factors influencing ITH. Notably, TDS maintained a stable effect across univariate and multivariate analyses, showing a negative association with ITH. In contrast, BRAF mutation exhibited a positive correlation with ITH in univariate analysis but a negative correlation in multivariate analysis. Then, we explored the potential reasons.

We first adjusted for any variables identified in univariate analysis and performed sensitivity analysis to explore the impact of changes in BRAF mutation. Adding any variables sequentially, the BRAF mutation maintained a positive association with ITH. Table 6 shows an example of BRAF mutation (β [95% CI]: 0.172 [0.117, 0.227], *p*  < 0.05) after adjusting for the number of positive lymph nodes. However, upon adjusting for TDS, the relationship reversed, showing a significant negative association between BRAF mutation and ITH (β [95% CI]: −0.084 [−0.13, −0.038], *p*  < 0.05, Table 6). These results suggested that TDS may be the factor causing the impact change of BRAF mutation on ITH in univariable and multivariable regression analyses.

**Table 6:** The association exploration between ITH and BRAF mutation by sensitivity analysis.

Variables	β [95% CI]	*p*
Adjustment 1		
Number of positive lymph nodes	0.054 [−0.029,0.136]	0.202
BRAF mutation	0.172 [0.117,0.227]	<0.001
Adjustment 2		
Number of positive lymph nodes	0.007 [−0.052,0.067]	0.810
TDS	−0.205 [−0.226,−0.185]	<0.001
BRAF mutation	−0.084 [−0.130,−0.038]	<0.001

*Note*: Adjustment 1 is to adjust one by one the variables that have a significant linear relationship with ITH except TDS. The sequence is lymph node ratio, number of positive lymph nodes, depth of tumor, length of tumor, T cells regulatory (Tregs), BRAF mutation, histological type (follicular), histological type (tell cell), T3 + T4, N1, advanced stage, postsurgical procedure assessment thyroid gland carcinoma status (yes), follow‐up radiation therapy (yes), extra thyroid carcinoma present extension status (minimal [T3]), extra thyroid carcinoma present extension status (moderate/advanced [T4a]), personal medical history (nodular hyperplasia), personal medical history (lymphocytic thyroiditis). Here we only show the first example of adjusting the Lymph node ratio. Adjustment 2 is to adjust all the above variables plus TDS, which only shows the TDS and lymph node ratio among the abovementioned variables.

Abbreviations: BRAF, v‐Raf murine sarcoma viral oncogene homolog B1; ITH, intratumoral heterogeneity; TDS, thyroid differentiation score.

**Figure 4:**
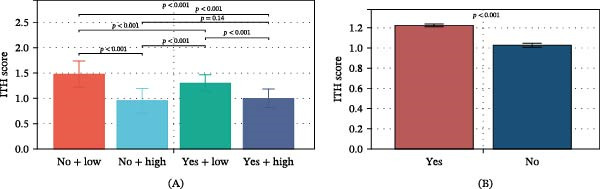
Comparison of ITH between different subgroups considering TDS and BRAF mutation. (A) Comparison of ITH among four subgroups (no + low: no BRAF mutation + low differentiation, no + high: no BRAF mutation + high differentiation, yes + low: BRAF mutation + low differentiation, yes + high: BRAF mutation + high differentiation). (B) Comparison of ITH between BRAF mutation and no BRAF mutation. Abbreviations: BRAF, v‐Raf murine sarcoma viral oncogene homolog B1; ITH, intratumoral heterogeneity; TDS, thyroid differentiation score.

Then, we divided patients into four groups based on BRAF mutation and TDS and compared ITH among groups. Subgroup analysis revealed the following results (Figure 4A): (1) ITH was significantly higher in the no BRAF mutation + low‐TDS group compared to the no BRAF mutation + high‐TDS group (*p*  < 0.05), indicating that low‐TDS increases ITH in the absence of BRAF mutation. (2) ITH was significantly higher in the BRAF mutation + low‐TDS group compared to the BRAF mutation + high‐TDS group (*p*  < 0.05), suggesting that low TDS also increases ITH in the presence of BRAF mutation. (3) ITH was significantly higher in the no BRAF mutation + low‐TDS group compared to the BRAF mutation + low‐TDS group (*p*  < 0.05), indicating that in cases of low TDS, the absence of BRAF mutation increases ITH. (4) No significant differences were observed between the no BRAF mutation + High TDS group and the BRAF mutation + high‐TDS group (*p*  > 0.05). When not considering the TDS, the BRAF mutation group had higher ITH (Figure 4B). Overall, these results supported the above findings that TDS has a stable effect on ITH, regardless of BRAF mutation status, while BRAF mutation had a negative effect on ITH when considering TDS.

The effect of TDS on the relationship between BRAF mutation and ITH was further explored by logistic regression analysis (dependent variable: high ITH vs. low ITH) and linear regression analysis (dependent variable: ITH score). The results showed that there was a negative correlation between BRAF and ITH when considering the TDS (OR < 1, β < 0, *p*  < 0.05, Table 7).

**Table 7:** Association among BRAF mutation, TDS combination, and ITH by logistic regression analysis and linear regression analysis.

Group	OR [95% CI]	*p*	β [95% CI]	*p*
No BRAF mutation + low TDS	Ref	Ref	Ref	Ref
No BRAF mutation + high TDS	0.020 [0.006,0.071]	<0.001	−0.522 [−0.620,−0.424]	<0.001
BRAF mutation + low TDS	0.211 [0.067,0.663]	0.008	−0.176 [−0.274,−0.079]	<0.001
BRAF mutation + high TDS	0.020 [0.004,0.088]	<0.001	−0.475 [−0.582,−0.369]	<0.001

*Note*: In logistic regression analysis, the dependent variable was high ITH and low ITH, and the OR value was calculated. In linear regression analysis, the dependent variable was the ITH score, and β was obtained.

Abbreviations: BRAF, v‐Raf murine sarcoma viral oncogene homolog B1; CI, confidence interval; ITH, intratumoral heterogeneity; OR, odds ratio; TDS, thyroid differentiation score.

Based on the above results, TDS affected the relationship between BRAF mutation and ITH. We further explored the role of TDS in BRAF mutation affecting ITH through interaction and mediation analysis. The interaction analysis results showed no interaction between TDS and BRAF mutations on ITH (*P* for interaction > 0.05, Table 8). The results of mediation analysis showed that the independent variable had a significant effect on the dependent variable (total effect, *p*  < 0.05), the independent variable had a significant impact on the intermediary variable (effect of BRAF mutation–TDS, *p*  < 0.05), and the intermediary variable had a significant effect on the dependent variable (effect of TDS–ITH, *p*  < 0.05). After adding the intermediary variable, the independent variable had a significant effect on the dependent variable (indirect effect, *p*  < 0.05), and the sign of the product of a and b is opposite to the sign of c’. Those results indicated that TDS mediates the effect of BRAF mutation on ITH through a suppression effect (Table 9).

**Table 8:** Logistic regression and interaction effect analysis on the association between BRAF mutation and ITH stratified by TDS level.

Variable	OR [95% CI]	*p*	*p* for interaction	
Overall	2.970 [1.890,4.680]	<0.001	—	
TDS group	—	—	0.068	
Low	0.210 [0.070,0.660]	0.008	—	
High	0.990 [0.300,3.300]	0.990	—	

Abbreviations: BRAF, v‐Raf murine sarcoma viral oncogene homolog B1; CI, confidence interval; ITH, intratumoral heterogeneity; OR, odds ratio.

**Table 9:** Mediation analysis among the BRAF mutation, TDS, and ITH.

Path	Coef	SE	*p*	CI [2.5%]	CI [97.5%]	Sig
BRAF mutation–TDS	−1.425	0.094	<0.001	−1.610	−1.24	Yes
TDS–ITH	−0.188	0.007	<0.001	−0.202	−0.173	Yes
Total	0.206	0.026	<0.001	0.155	0.258	Yes
Direct	−0.098	0.021	<0.001	−0.139	−0.056	Yes
Indirect	0.304	0.026	<0.001	0.255	0.353	Yes

Abbreviations: BRAF, v‐Raf murine sarcoma viral oncogene homolog B1; CI, confidence interval; ITH, intratumoral heterogeneity; SE, standard error; TDS, thyroid differentiation score.

### 3.4. Potential Mechanisms Underlying ITH’s Prognostic Impact in PTC

**Figure 5:**
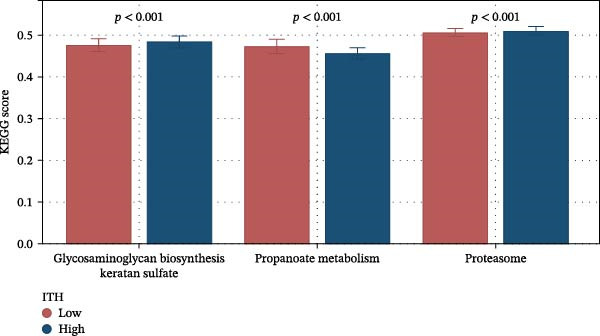
Comparison of the KEGG score of the key pathways between the low‐ITH group and the high‐ITH group. Abbreviations: ITH, intratumoral heterogeneity; KEGG, Kyoto Encyclopedia of Genes and Genomes.

**Figure 6:**
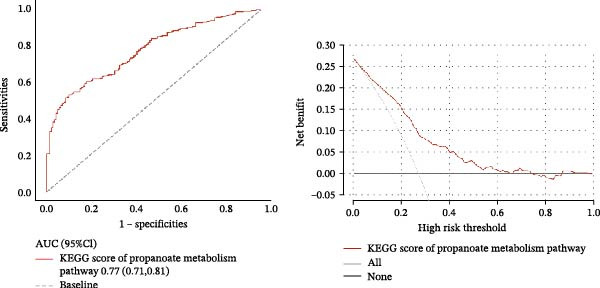
Prediction efficiency evaluation of the propanoate metabolism pathway score on ITH. (A) ROC curves of ITH according to the KEGG score of propanoate metabolism. (B) DCA curves of ITH according to the KEGG score of propanoate metabolism. Abbreviations: AUC, area under the curve; CI, confidence interval; DCA, decision curve analysis; ITH, intratumoral heterogeneity; KEGG, Kyoto Encyclopedia of Genes and Genomes; ROC, receiver operating characteristic curve.

Given the significant impact of ITH on prognosis, we investigated the underlying mechanisms. Using ssGSEA, we calculated pathway scores for 186 KEGG pathways. Comparison of high‐ and low‐ITH groups revealed significant differences in KEGG scores for 126 pathways (*p*  < 0.05), of which 89 were highly significant (*p*  < 0.001). Correlation analysis identified that only three pathways (glycosaminoglycan biosynthesis keratan sulfate, propanoate metabolism, and proteasome) were significantly associated with ITH (*p*  < 0.05). Glycosaminoglycan biosynthesis keratan sulfate pathway and proteasome pathway were upregulated in the high‐ITH group, indicating pathway activation, while the propanoate metabolism pathway was downregulated, suggesting suppression (Figure 5). Multivariate linear regression confirmed that only the propanoate metabolism pathway was independently associated with ITH, highlighting its pivotal role (Table 10). ROC analysis showed that the propanoate metabolism pathway predicted ITH with an AUC of 0.77 (Figure 6A), with sensitivity, specificity, and accuracy of 0.53, 0.90, and 0.65, respectively. DCA demonstrated that propanoate metabolism provided a clinical net benefit for predicting ITH within a threshold probability range of 0.02–0.61 (Figure 6B). These results highlighted the importance of the propanoate metabolism pathway on ITH.

**Table 10:** Multivariable linear regression analysis on the key variables associated with ITH.

Variable	β [95% CI]	*p*
Glycosaminoglycan biosynthesis keratan sulfate	1.035 [−0.142,2.212]	0.086
Propanoate metabolism	−2.123 [−3.231,−1.015]	<0.001
Proteasome	0.649 [−1.031,2.329]	0.449
TDS	−0.189 [−0.208,−0.169]	<0.001
BRAF mutation	−0.103 [−0.144,−0.063]	<0.001

Abbreviations: BRAF, v‐Raf murine sarcoma viral oncogene homolog B1; CI, confidence interval; ITH, intratumoral heterogeneity; TDS, thyroid differentiation score.

## 4. Discussion

PTC is a common endocrine tumor, accounting for ~80% of THCAs. Its prognosis is influenced by various factors, with ITH being one of the key determinants. In this study, we identified that ITH serves as a risk factor for poor DFI in patients with PTC. Furthermore, we observed that TDS and BRAF mutations impacted ITH, with TDS attenuating the effect of BRAF mutations on ITH.

BRAF is one of the most common driver mutation genes in PTC. The BRAF V600E mutation activates the MAPK signaling pathway, triggering a series of molecular and cellular events that influence ITH. In our study, we found a positive correlation between BRAF mutation and ITH. The BRAF V600E mutation, by continuously activating the MAPK signaling pathway, promotes cell proliferation, survival, and migration. This persistent signaling activation not only enhances the aggressiveness of tumor cells but may also induce the formation of different clonal subpopulations, thereby increasing the internal heterogeneity of the tumor [16, 17]. Additionally, the mutation has been shown to increase DNA damage and genomic instability due to the overactivation of the MAPK pathway, which induces reactive oxygen species (ROS) accumulation, leading to DNA damage and chromosomal aberrations, further contributing to the genetic diversity of tumor cells [18]. BRAF mutation can also modulate key factors in the tumor microenvironment, such as cytokines, chemokines, and growth factors, thereby influencing ITH. For example, the BRAF V600E mutation promotes the secretion of pro‐inflammatory factors like VEGF and IL‐6, facilitating tumor angiogenesis and immune evasion. These factors create adaptive ecological niches for different tumor cell subpopulations, further aggravating ITH [19].

Tumor differentiation level reflects the degree to which cancer cells retain the characteristics of their cell of origin. In PTC, the differentiation level is evaluated by the TDS. Our study found a negative correlation between TDS and ITH. This might be because, in poorly differentiated tumors, cancer cells tend to lose the functional characteristics of thyroid follicular cells, such as iodine uptake and thyroid hormone synthesis. These cells often exhibit higher genomic instability and greater adaptability, resulting in the formation of more genetic and epigenetic clonal subpopulations, thereby increasing ITH [20]. Reduced differentiation is also associated with enhanced resistance to radioactive iodine (RAI) therapy. Resistance to RAI therapy is considered an important manifestation of ITH, as tumor cells with varying degrees of differentiation exhibit different sensitivities to treatment. Tumors with lower differentiation levels are less sensitive to RAI, leading to increased heterogeneity among residual tumor cells after treatment [21, 22]. Poorly differentiated tumor cells often exhibit metabolic reprograming, such as alterations in propionate metabolism, glycolysis, and oxidative phosphorylation. This metabolic reprograming not only enhances tumor cell survival [23] but also provides multiple survival advantages for different clonal subpopulations, exacerbating ITH [24].

Moreover, our findings suggest that TDS can obscure the influence of BRAF mutation on ITH. Highly differentiated thyroid tumors (high TDS) typically retain functional characteristics of thyroid follicular cells, such as iodine uptake and thyroid hormone synthesis [25]. These tumor cells exhibit higher genomic stability, with lower probabilities of genetic mutations and chromosomal aberrations, thereby limiting the contribution of BRAF mutation to ITH. Conversely, poorly differentiated thyroid tumors (low TDS) often display significant genomic instability, including defects in DNA repair and chromosomal instability (CIN). This genomic instability amplifies the effects of BRAF mutation, leading to more clonal variations and increased ITH. In highly differentiated tumors, a relatively stable genomic background may counteract or limit the effect of BRAF mutation in promoting ITH. Therefore, the influence of BRAF mutation on ITH is masked by the stabilizing effect of differentiation level [20]. From a metabolic perspective, the metabolic stability of highly differentiated tumor cells provides a consistent energy supply while limiting metabolic flexibility, reducing clonal diversity. Poorly differentiated tumors exhibit significant metabolic reprograming, such as increased glycolysis, lactate metabolism, and lipid metabolism. This metabolic adaptability offers more survival advantages and selective pressures for tumor cells, promoting ITH. The metabolic characteristics of highly differentiated tumors may limit BRAF mutation‐induced metabolic reprograming, thereby reducing the contribution of BRAF mutation to ITH [22]. Furthermore, highly differentiated thyroid tumors often maintain high expression levels of thyroid‐specific genes, which may regulate the function of BRAF mutation. For example, high expression of sodium‐iodide symporter (NIS) enhances iodine uptake in tumors, whereas BRAF mutation has been shown to suppress NIS expression [26, 27]. This interaction may be influenced by differentiation levels. Thyroid peroxidase (TPO) and dual oxidase (DUOX), which are associated with ROS metabolism, may limit BRAF mutation‐induced oxidative stress under conditions of high differentiation [26], thereby inhibiting the increase in ITH [28, 29].

Finally, the pathway analysis revealed that the propionate metabolism pathway is most strongly associated with ITH, suggesting that tumor energy metabolism processes in PTC drive the essence of ITH. As a crucial metabolic pathway, the propionate metabolism pathway plays a pivotal role in tumor metabolic reprograming. Propionate metabolism interacts with key enzymes in cellular energy metabolism, such as pyruvate dehydrogenase (PDH) and acetyl‐CoA carboxylase (ACC), enhancing the metabolic adaptability of tumor cells. Tumor cells typically rely on metabolic reprograming to adapt to microenvironmental limitations, including restricted oxygen and nutrients, as well as the accumulation of acidic metabolites. Propionate, as a metabolic byproduct, regulates energy balance in tumor cells by modulating pathways such as AMPK (5^′^ AMP‐activated protein kinase), facilitating tumor proliferation and metastasis. Under hypoxic conditions, energy generated through the propionate metabolism pathway supports cell survival and influences cytokine production and gene expression, enabling tumor cells to evade immune surveillance [30]. This metabolic adaptability and plasticity allow tumor cells to maintain heterogeneity within the complex microenvironment, promoting tumor invasiveness and metastasis [31, 32]. Propionate may also enhance the survival of mutated cells by modulating genomic instability, further increasing genetic heterogeneity. This metabolic regulation‐driven accumulation of mutations leads to genetic differences among subclonal populations, further exacerbating ITH [22]. Specifically, in tumor cells, increased propionate flux results in the accumulation of intracellular propionyl‐CoA and its metabolite, methylmalonic acid (MMA). On one hand, propionyl‐CoA acts as an “acyl donor” that significantly enhances histone lysine propionylation (Kpr) and propagates to nonhistone proteins (such as p53 and DNA‐PK). This process remodels chromatin accessibility and transcriptional networks via an imbalance between p300/CBP and sirtuins, resulting in the downregulation of high‐fidelity DNA repair and apoptosis while upregulating cell cycle progression and stress survival programs [33, 34]. On the other hand, excessive propionyl‐CoA and related metabolites cause CoA “trapping” within mitochondria, suppress the respiratory chain, and elevate ROS levels, thereby inducing replicative stress and DNA double‐strand breaks. This increases the mutational burden and chromosomal aberrations at their source [35]. Meanwhile, MMA can promote epithelial–mesenchymal transition (EMT), drug resistance, and matrix remodeling through pathways such as TGF‐β → SOX4, enhancing the survival and dissemination potential of damaged clones [36]. In summary, metabolites derived from propionate metabolism contribute to mitochondrial dysfunction and elevated ROS, which trigger replicative stress and DNA damage. These effects drive mutations in both mitochondrial and nuclear DNA, as well as CIN. Subclones carrying distinct mutational profiles are selectively retained and gradually occupy ecological niches, fostering genomic instability and thereby increasing tumor genetic heterogeneity.

The innovation of this study lies in the integration of BRAF mutation, TDS, and propionate metabolism pathway within a unified framework to systematically analyze their roles in the ITH of PTC. However, the study also had some limitations. Firstly, we did not do experiments to verify due to the limitations of the conditions. Secondly, we did not use data from other centers to verify our conclusions. We will collect related data for verification in the future.

## 5. Conclusion

Our findings suggest that ITH can serve as an important prognostic marker for PTC patients. The BRAF mutation and TDS were significantly associated with ITH, but TDS had a suppressive effect on the impact of the BRAF mutation on ITH. Furthermore, the dysfunction of the propionate metabolism pathway was found to be associated with ITH, suggesting that the pathway provides energy support and metabolic adaptability for tumor heterogeneity. Our findings offer new research directions for personalized PTC treatment and precision medicine.

## Author Contributions

Ying‐er Feng contributed to the study conception and design. Ying‐er Feng and Cun‐jie Wang contributed to the collection, assembly, analysis, and interpretation of the data.

## Funding

The authors received no specific funding for this work.

## Disclosure

All authors wrote and approved the final manuscript.

## Ethics Statement

The Ethics Committee of The Second Affiliated Hospital, Zhejiang University School of Medicine, agreed to submit the study for review and has waived the need for ethical approval.

## Consent

The authors have nothing to report.

## Conflicts of Interest

The authors declare no conflicts of interest.

## Data Availability

The data will be made available upon request.
